# Cancer Pain Management in French-Speaking African Countries: Assessment of the Current Situation and Research Into Factors Limiting Treatment and Access to Analgesic Drugs

**DOI:** 10.3389/fpubh.2022.846042

**Published:** 2022-03-18

**Authors:** Yacine Hadjiat, Serge Perrot

**Affiliations:** ^1^Inserm (National Institute of Health and Medical Research, U987), Paris-Saclay University, Paris, France; ^2^Inserm (National Institute of Health and Medical Research, U987), Paris, France; ^3^Pain Management Department, Cochin Hospital, Paris Cité University, Paris, France

**Keywords:** cancer, pain, Africa, French-speaking, opioid, policy

## Abstract

**Introduction:**

There is a dearth of research on the incidence and treatment of cancer pain in Africa. Yet Africa, with other developing countries, accounts for more than half of all cancer diagnoses, and it is estimated that cancer incidence in Africa will double by 2030.

**Objectives:**

This research protocol outlines an approach to investigate cancer pain in French-speaking African countries. The protocol intends to determine and describe the treatment and management of cancer pain in these countries. Barriers to treating cancer pain will be explored and the results will be collated to make a series of recommendations on policy positions, regulatory frameworks and protocols.

**Methods:**

A mixed-methods, co-creation methodology has been selected to ensure the societal impact of the research outcomes. This research will use both qualitative and quantitative data collection methods and analyses. The research will begin with a review of the policies and legislation that exist in relation to cancer pain management and the use of analgesics, in each French-speaking African country. An Experts Steering Committee will then be created to provide guidance on the protocol and research design and access to participants, as well as to execute on the administration of surveys to local structures and international experts. A series of semi-structured, qualitative interviews with experts and clinicians in the field of screening and management of cancer pain and access to treatment will follow. Purposive and snowball sampling will be used to select the respondent experts. The semi-structured interviews will be conducted to determine the main trends and barriers to the treatment of cancer pain in French-speaking African countries. From this qualitative research, two surveys will be developed and then administered: one to validate the policy and regulatory context, and the other to determine experts and healthcare professionals experience and perceptions of cancer pain.

**Results/Conclusions:**

The results will be analyzed using quantitative and qualitative methods to determine themes and perceptions of cancer pain and treatment, from the policy level to the healthcare professional level. Evaluation of the results will lead to recommendations for a comprehensive framework for cancer pain treatment in French-speaking Africa.

## Introduction

The incidence and impact of cancer is increasing in the developing world. More than 57% of all cancer patients are estimated to be in Africa, Asia and Latin America (1, 2); with these regions accounting for 65% of cancer deaths, and 48% of 5-year prevalent cancer cases (3). The incidence of cancer in African regions is set to double between 2012 and 2030 (3). GLOBOCAN reports the highest incidence of cancer in Southern and Northern Africa, followed by Western Africa (4).

**Figure 1:**
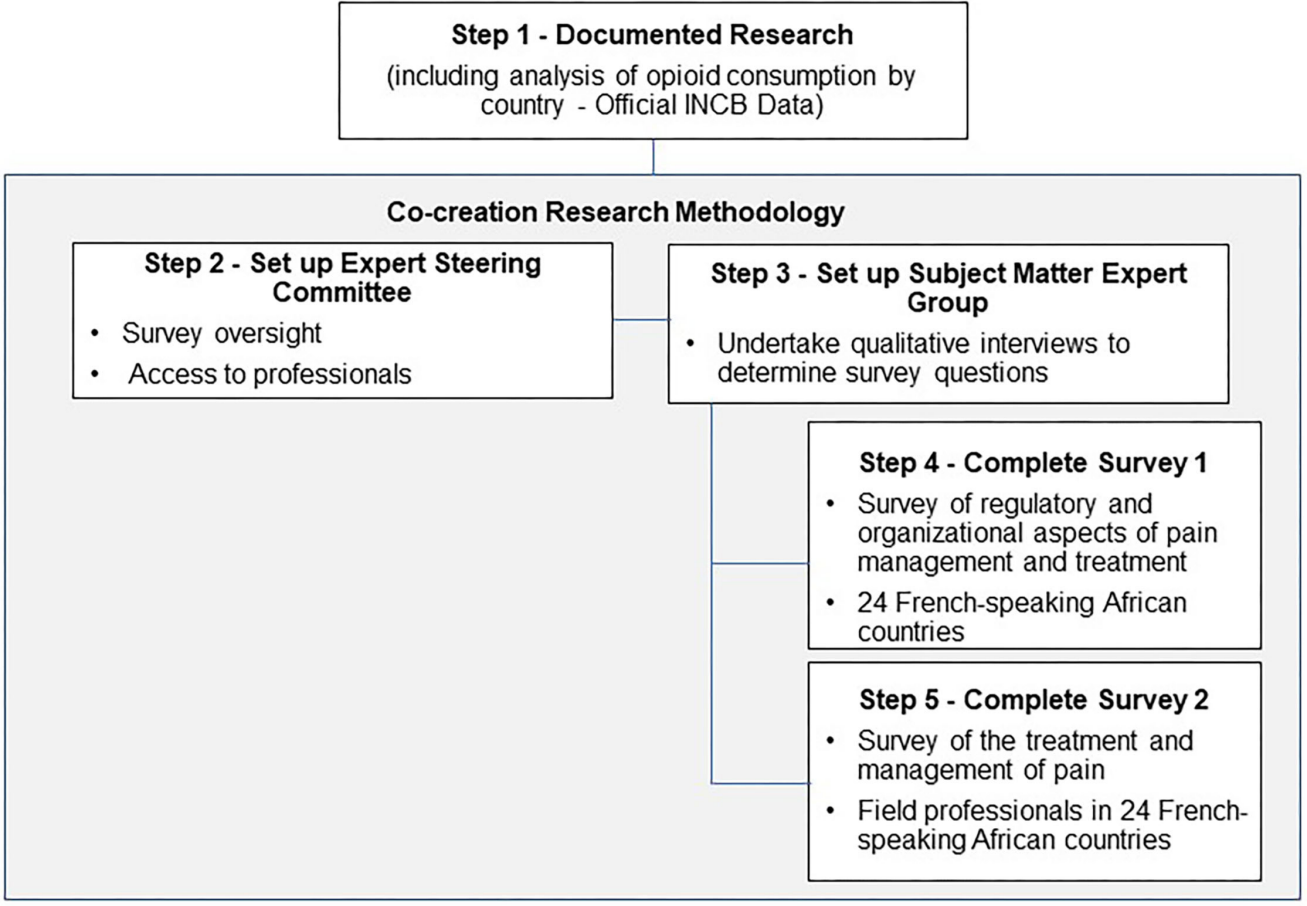
Components of the research process.

Updated statistics for French-speaking countries in Africa are not available in the literature. There is a disproportionate focus on Sub-Saharan Africa (mainly English-speaking) with little to no data available for other regions within Africa. The American Cancer Society's Cancer Atlas provides updated information on the incidence and mortality rates from cancer in sub-Saharan Africa which shows an increasing incidence of prostate and cervical cancer (5). Cancers associated with the adoption of a western lifestyle are prevalent (6), as are those associated with bacterial and viral infections; the latter being the most common type of cancer, and leading cause of cancer death (7). Added to the burden of care created by an increasing cancer prevalence rate, is the social, economic and personal burden of treating the pain associated with cancer. The components of the research process are depicted in Figure 1.

Two out of three patients with cancer suffer from moderate to severe pain in the advanced stages of their disease (8). Pain is ranked as one of the top four non-communicable diseases world-wide, yet the barriers to effective treatment and care remain significant, especially in developing countries (9). Pain creates a major public health problem due to the large number of patients suffering from it, but also because of the physical (reduced quality of life), emotional (isolation, anxiety, depression, sleep disorders), and economic (work stoppages, consumption of care resources, hospitalization, healthcare expenditure and disability compensation) (10) issues it raises when not sufficiently managed (10–12). One review study found pain to be associated with lower socio-economic status and reduced economic activity, adding an additional burden to developing countries (13).

### Cancer Pain in the Developing World

According to the WHO, 83% of the world's population (5.5 billion people) have inadequate access to treatment for moderate to severe pain and little or no access to controlled drugs (8). In addition, the pathophysiology of cancer is not homogenous-pain may be as a result of the cancer, or the treatment (14). The frequency and multiple causes of cancer pain make its treatment and management more difficult, especially in resource constrained developing countries. Table 1 provides a summary of some of the main barriers to pain treatment in developing countries. It is clear that access to pain treatment is a multi-faceted problem covering medical and patient rights, duty of care, ethics, regulation and policy frameworks and access to resources.

**Table 1:** Barriers to the optimal treatment of pain in developing countries (15).

**Structural and knowledge barriers**	**Socio-cultural and attitude barriers**	**Economic barriers**
• Pain is not recognized as a public health priority •General Practitioners do not have the required knowledge and expertise to treat pain •There is a scarcity of qualified pain specialists •Many schools of medicine and of pharmacy have very limited time included in their curricula for the treatment of pain •Resources and medical personnel are unevenly distributed •Administrative requirements to access pain medications are significant and deter usage •Regulation of pain medication may be restrictive at a country level due to fears of misuse, abuse and diversion	• Cultural beliefs impact the understanding and treatment of pain •Many accept pain as a natural and unavoidable part of aging and disease •Traditional medicines are the preferred treatment in some countries •Religious beliefs in some countries see pain as a part of atonement and this leads to reduced help-seeking behavior and treatment •Patients, healthcare workers and governments may have a fear of using opioids for treatment	• Competing demands for resources at a country level impact negatively on healthcare spending in general, and on the treatment of pain specifically •The cost of medicines and other treatments could be high or not seen as priority •Vulnerable sub-groups and people with lower socio-economic status have less access to resources to seek and pay for treatment

### Access to Essential Pain Treatments

Opioid analgesics, listed as essential medicine by the WHO, are considered the safest, most effective and cost-effective treatment for moderate to severe cancer pain. Access to this treatment remains a problem in developing countries, intensifying the negative impact to quality of life, levels of suffering and ability to work (16).

There is a dearth of research on treating pain in Africa. Research in developing Asian countries has found that cancer pain is under-treated; 59% compared to 39% in the United States (17). As a specific example, the ACHEON Study surveyed cancer patients and physicians across 10 Asian countries and found that 86% suffered from moderate to severe pain, 1 in 3 patients was not satisfied with their pain treatment, and only 53% of patients were treated with opioids (18).

The limited prescription of opioids in developing countries vs. developed ones may be due to concerns relating to dependence, and many countries have legislation regulating their use (19, 20). Cleary et al. (21) conducted a systematic study of the availability and accessibility of opioids for the management of cancer pain across the African continent. They surveyed 25 of 52 countries and found that many countries had severely restricted formularies of opioids and only 15 of 25 had morphine available in oral immediate release, controlled release and injectable formulations. Findings also indicated that even when opioids are on formulary, they are often unavailable. The authors conclude that Africa remains at the lowest levels of opioid consumption globally, attributable to low access, low levels of health care worker training, and outdated regulation and policy (21). There is some indication that opioid prescription has increased (11) although there is a lack of data collating recent opioid prescription levels across African countries. The current research protocol aims to determine the obstacles to managing cancer-related pain, using opioid analgesics, in French-speaking Africa. This is especially necessary as Human Rights Watch (22) notes French-speaking African countries have limited access to palliative care and limited access to opioids in comparison to English-speaking African countries, while the population is expected to rise for people over the age of 65 years.

The following objectives guide the development of this protocol. The research aims to:

a) Describe the perception of the current status of cancer pain management in the different countries in French-speaking Africa.
b) Document the consumption of opioids across French-speaking African countries.
c) Determine healthcare workers experience of the availability of strong opioids and associated regulation in cancer pain in the different countries of French-speaking Africa.
d) Outline the different factors that may limit the availability of cancer pain treatments and care in the different countries of French-speaking Africa.
e) Propose recommendations for improvement, especially in the areas of education on cancer pain management and palliative care.

## Methods

This research will use a mixed methods research methodology, including both qualitative and quantitative data collection methods and analyses. A systematic review of the policy and regulatory environment will be conducted, followed by semi-structured interviews and surveys. The International Narcotics Control Board (INCB) will be collaborating on this research project and will be providing the official data of opioid consumption by country from 1999 to 2018. Quantitative analysis of opioid consumption by country will be analyzed from the INCB data set for the selected countries.

Survey research will be conducted to determine the perception of levels of opioid prescription in the treatment of cancer pain in French-speaking Africa. In this research protocol, 24 French-speaking countries where French is the first or second language will be selected (see Table 2) (23).

**Table 2:** French-speaking countries in Africa which will be used in the survey.

**Democratic Republic of Congo**	**Mali**	**Benin**	**Equatorial Guinea**
Madagascar	Senegal	Togo	Comoros
Cameroon	Chad	Central African Republic	Seychelles
Ivory Coast	Guinea	Republic of the Congo	Morocco
Niger	Rwanda	Gabon	Algeria
Burkina Faso	Burundi	Djibouti	Tunisia

### Process of Research

The research method integrates an assessment of the current state and evaluation of factors limiting cancer pain management and treatment access. It will involve national stakeholders, politicians, public health agencies and international organizations such as WHO, the International Narcotics Control Board (INCB), the International Association for the Study of Pain (IASP), and health care providers in the field.

The research will be guided by an Expert Steering Committee convened by the research team. Qualitative semi-structured interviews will be held with experts and field professionals across countries in French-speaking Africa and internationally.

A survey focused on regulatory and organizational aspects of pain management and treatment will be conducted in the identified French-speaking countries.

Finally, a survey of field professionals involved in the management of pain will be undertaken.

The research team will develop the instruments and analysis plan, letters of consent, and ethical considerations in a protocol to be considered by a suitable medical ethics committee.

### Components of the Research

#### Research on Policy and Legislative Frameworks

The research will begin with an extensive desktop review of the policies and legislation that exist in relation to cancer pain management and the use of opioids in each French-speaking African country. This will form the baseline against which results will be compared. In addition, a list of suitable WHO and related guidelines on cancer pain treatment (specifically including the WHO Essential Medicines List), and gold standard policy and legislation will also be documented to form a benchmark against which participant's perceptions will be compared.

#### Data Analysis of Country Level Opioid Consumption

Existing historical data from the INCB will be analyzed descriptively. Data is available across the different countries form 1999-2018. Frequencies and change trends will be calculated. This information will form the basis for country comparisons from the surveys and interviews. Opioid analgesic consumption will be linked to cancer diagnoses in the INCB data by cross checking INCB numbers with cancer epidemiology data. It is acknowledged that INCB returns do not distinguish between opioid use for cancer pain and use for other indications. This is noted as a limitation of the study. Further, it is likely the INCB data is impacted by the biases inherent in large data collation databases of this nature, with the limitations of data respondent biases and missing data acknowledged. For this reason, this data will be used in a general sense for insights and trend analysis.

The research on policy and legislative frameworks, and the data analysis of country level opioid consumption will be conducted by the researchers and does not form part of the co-creation research methodology. Following this research and analysis, an Experts Steering Committee will be created, and will drive the co-creation of the research using the steps below.

#### Creation of Experts Steering Committee

An Experts Steering Committee will be created including national and international experts from the scientific community. It is essential that this committee is representative of, and connected to, French-speaking African countries in order to ensure access to all 24 targeted countries. Therefore, the selection criteria for the committee will include subject-matter expertise, connections to/relationships with international health organizations and African country institutions and governments. The steering committee will provide guidance and access and execute on the administration of the surveys to local structures and international experts. In addition, the steering committee will approve the criteria required for each survey, such as the determinants of cancer diagnosis, the definition of healthcare worker, and the definition of pain treatment beyond medical intervention. The committee will provide expert opinion on the content of all surveys before administration and the study authors will coordinate this process.

The co-creation of the survey content is an important component of the research, and the collective knowledge and experience of the Committee will be utilized to create relevant and up-to-date surveys. van Dijk-de Vries et al. (24) assert that the active participation of stakeholders in health research practice is important to generate societal impact of outcomes. A co-creation approach involves stakeholders as full and equal partners in all phases of the research process and leads to outcomes that are more likely to be acceptable, valuable, and enduring than traditional research approaches (24). The co-creation process will also promote a sense of ownership and collaboration within the research project, and this is considered preferable to using a survey that is designed by the study authors before the research commences.

An important consideration in survey development and design is bias. Each survey will be designed based on the findings of the semi-structured interviews with experts (see Section Creation of Experts Steering Committee). Before administration, each survey will be piloted for item suitability and potential social desirability bias. In addition, as each survey is administered, a cross range of participants will be chosen to ensure variability in perspectives.

#### Semi-Structured Interviews of Experts

The study will then undertake a series of semi-structured, qualitative interviews with experts and clinicians in the field of screening and management of cancer pain and access to treatment. The development of the semi-structured interviews will follow the rigorous process espoused by Kallioet al. (25) to ensure an objective and trustworthy design.

Purposive sampling will be used to select the respondent experts. These participants will be selected by invitation heads of local and regional Pain and Palliative Care medical organizations familiar to the researchers). Snowball sampling (26) will then be used as the heads of these units cascade the request to their members and networks. The experts will be chosen based on their positions in relevant pain organizations. One pain and one palliative care expert from each country will be approached. These experts will then be asked to cascade the request to at least 2 experts in their countries. A total sample size of 72 experts is thus planned, however new respondents will be recruited until saturation is achieved.

The semi-structured interviews will be guided by the Barriers to Pain Treatment Framework illustrated in Table 1. The three thematic areas of Structural and knowledge barriers; Socio-cultural and attitude barriers, and Economic barriers will form the basis of the interview schedule. A thematic analysis (27) of the content of the semi-structured interviews will then be conducted with the aim of identifying key content themes and validating the Barriers Framework to determine suitability, comprehensiveness and relevance. Thematic analysis “involves the identification of themes with relevance specific to the research focus, the research question, the research context and the theoretical framework. This approach allows data to be both described and interpreted for meaning” (p.1) (27). The revised framework will be further validated by the Expert Steering Committee before the final Barriers to Pain Treatment Framework is adopted.

The interviews will be conducted remotely using digital channels such as video and voice conferencing. The subject-matter experts will be asked to consent to the video or voice call being recorded and the content of the recording will be kept confidential. The recording will be transcribed by a research assistant, and checked by a second assistant for reliability, thoroughness and correctness (27). This transcription will form the basis for the analysis and the voice recording will be deleted.

Once the thematic analysis of the semi-structured interviews has been used to inform the Barriers to Pain Treatment Framework in Table 1, the Framework will be used as a reference for developing the two surveys: the first survey will focus on organizational aspects, and the second survey will focus on field and care aspects. As this revised Framework needs to be developed and validated, the content and structure of the surveys below are an indication of expected format, which may be adapted based on the results of semi-structured interviews.

It will not be possible to control for the levels of expertise and experience of the interviewees, nor for their demographic characteristics (such as age and gender). Any of these variables may act as confounding variables and this will need to be noted as a limitation of the study.

#### Organizational Survey

The organizational survey will take the form of a structured questionnaire, including both closed and open-ended questions. The intent is to validate data on the policy and legislative framework for pain treatment in each country. Apart from providing a view of the characteristics and level of enablement each policy and regulatory environment provides, this survey will also enable each country to draw up an updated inventory of cancer pain management structures and the local organizational, regulatory, and legislative context. The survey will consist of 5 parts:

- National organizational context.
- Legislative and regulatory context.
- Education and training on pain and palliative care.
- Organization of pain management.
- Expert summary of the current situation regarding opioid-based cancer pain management at the national level.

A country level liaison will be selected for each country. The Expert Steering Committee will assist in this determination and allocation. The criteria for inclusion as a country liaison include working within the public pain management system, suitable level of seniority to ensure access to policy and legislative materials, expertise in the application of legislation and guidelines in the area of pain treatment and intervention.

Each survey will be provided electronically. The results will be collated by the research team and coded for country and respondent. Respondent written consent will be obtained, and this information will be kept confidential.

A coding and analysis structure will be developed by the research team and approved by the Expert Steering Committee. This structure will include international best practice guidelines on the policy and legislative framework necessary for pain treatment. The structure will be based on the WHO Guideline on ensuring balanced national policies for access and safe use of controlled medicines, and related guidelines associated with this article (28). A full list of guidelines will be developed, and will include the WHO list of essential medicines for cancer pain (current indications range from 36% use of the list of essential medicines to almost 80% depending on the country) (29). The analysis will be primarily qualitative, but quantitative frequencies across countries will be possible when analyzing by features of each national policy and legislative space.

#### Field and Care Survey

The second survey will be directed at practitioners involved in the management of cancer pain, collecting field data on practices, and identifying the main barriers to access to opioid analgesics and potential levers to improve this use. The practitioners will be identified by each country liaison and/or the subject matter expert group. A representative sample of healthcare professionals from various settings will be targeted and contacted via the members of the Scientific Committee and local learned societies or associations. Thirty participants for each country will be selected against the criteria of working in the field of pain management, diverse locations, and varying contexts. The subject matter expert group will approve the participants.

This survey will consist of 5 parts:

- Demographic data.
- Cancer pain management in your practice.
- The current situation regarding pain management.
- Barriers to access to opioid analgesics to manage pain.
- Solutions to improve cancer pain management.

The data will be collected electronically, coded and kept confidential. Written consent will be obtained. No personal link to the respondents will be available in the final database.

The objective is to identify the needs and expectations of healthcare professionals, mainly in managing cancer related pain, with a specific focus on the use of opioids. The data will be analyzed against the revised pain treatment framework outlined in Section Data Analysis of Country Level Opioid Consumption. Quantitative rating scales will be analyzed for frequencies of responses and will be compared across countries.

The same limitations for the interview respondents apply to the respondents of both surveys. Lack of ability to control for the levels of expertise, experience and demographic characteristics of survey respondents may result in confounding variables that impact the results. This will be noted as a limitation of the study.

### Institutional Collaborations to the Research Project

This research will be possible as a result of collaboration with teams of the INSERM Unit (U987) in the qualitative analysis and exploitation of databases. In addition to the partnership with the Research Team on the Physiology and Pharmacology of Pain (Inserm U987) set up for the supervision of this research, a collaboration with the IASP (International Association for the Study of Pain), the French Society for the Study and Treatment of Pain (SFETD), the WHO, the INCB and Pain without Borders (DSF) will take place.

## Anticipated Results

### Expected Results

Current public health policies emphasize opioid-related risks, including the opioid epidemic in North America. Conversely, the lack of opioid treatment for cancer pain and palliative care in developing countries is not addressed because data is scattered, and factors are poorly analyzed. Such a study would make it possible to put in place proposals adapted to the medical and political context, aimed at public health officials, to help doctors improve the quality of life of cancer patients while minimizing the risks.

In 2019 the scientific community, particularly in oncology and palliative care, evoked the concept of “The other opioid crisis”—the crisis of access to care and analgesic drugs (12). This research will shed more light on this ethical, political, and scientific issue, study its causes and identify corrective actions. The overarching goal is to enable all patients to have access safely to pain relief as a fundamental human right, as stipulated by the United Nations and the Montreal Declaration 2010.

### Publication Plan

Four publications are planned in order to report on the progress of the project and the results obtained at each of the major stages of the research:

#### Publication 1

An original article in a peer-reviewed scientific journal will be published using the data from the semi-structured interviews and the results of the associated qualitative analysis. This will allow the researchers to develop the two surveys by identifying the areas to be explored.

#### Publications 2 and 3

Original articles in peer-reviewed scientific journals will be published from the results of the two surveys of (i) managers (Organizational) and (ii) caregivers (Field and Care), covering the status of cancer pain management in French-speaking African countries and identification of limiting factors for opioid use.

#### Publication 4

Finally, a publication collating the findings will be published with the intent of providing a view of cancer pain in French-speaking Africa, outlining the main factors that limit the management and use of opioid analgesics and proposed necessary improvements.

## Discussion

The paucity of research on cancer pain levels, treatment and management in French-speaking Africa (22) suggests that more work is required to understand the context and nature of untreated and under-treated cancer pain. This research will contribute toward the literature on cancer pain, enabling evidence-based decisions on policy and regulation, as well as the provision of effective treatment. With “the other opioid crisis” identified in developing countries, it is essential to understand the levels of cancer pain and barriers and challenges to treatment in order to implement protocols for effective pain management that suit the policy environment, medical professional and patient. This research aims to collate the results into an evaluative statement and recommendations for effective cancer pain treatment and management practices that consider local conditions and resources.

## Data Availability Statement

The original contributions presented in the study are included in the article/supplementary material, further inquiries can be directed to the corresponding author/s.

## Author Contributions

All authors listed have made a substantial, direct, and intellectual contribution to the work and approved it for publication.

## Conflict of Interest

The authors declare that the research was conducted in the absence of any commercial or financial relationships that could be construed as a potential conflict of interest.

## Publisher's Note

All claims expressed in this article are solely those of the authors and do not necessarily represent those of their affiliated organizations, or those of the publisher, the editors and the reviewers. Any product that may be evaluated in this article, or claim that may be made by its manufacturer, is not guaranteed or endorsed by the publisher.
